# Facile growth of carbon nanotubes using microwave ovens: the emerging application of highly efficient domestic plasma reactors

**DOI:** 10.1039/c9na00538b

**Published:** 2019-10-17

**Authors:** Yang Liu, Naishun Guo, Pengfei Yin, Chao Zhang

**Affiliations:** Department of Biomedical Engineering, Sun Yat-sen University Guangzhou China 510006 liuyang56@mail.sysu.edu.cn zhchao9@mail.sysu.edu.cn

## Abstract

The facile growth of carbon nanotubes (CNTs) using microwave radiation reveals a new way for the cost-effective synthesis of CNTs for a wide range of applications. In this regard, domestic microwave ovens can be used as convenient plasma reactors to grow CNTs in a very fast, simple, energy-saving and solvent-free manner. The special heating mechanism of microwaves can not only accomplish the fast growth of high-density CNT brushes within tens of seconds, but also eliminate the need for a flammable gaseous carbon source and an expensive furnace. By carefully selecting the substrate and catalyst, low-temperature growth of CNTs can also be achieved on low-melting point organic polymers at atmospheric pressure. Highly localized heating near the catalyst nanoparticles was observed under microwave irradiation, and this phenomenon can be utilized to grow CNTs at desired locations on the substrate to fabricate CNT-based nanoelectronics *in situ*. Finally, the microwave growth of CNTs is highly adaptive to different carbon sources, substrates and catalysts, showing enormous potential to generate functionalized CNT-based composites for emerging advanced applications.

## Abstract 2

Carbon nanotubes are obtained using a domestic microwave oven in a few seconds at room temperature and atmospheric pressure.

## Graphical abstract

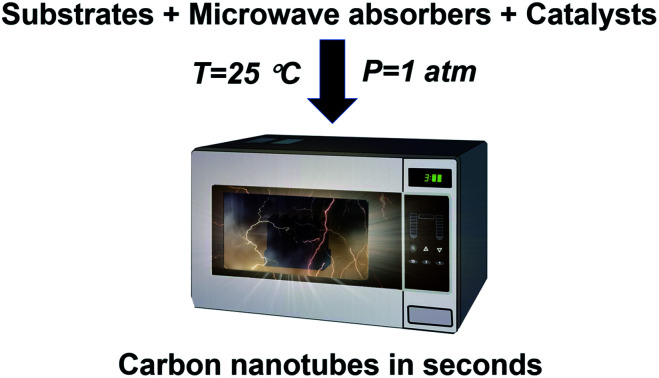

## 1. Introduction

**Fig. 1:**
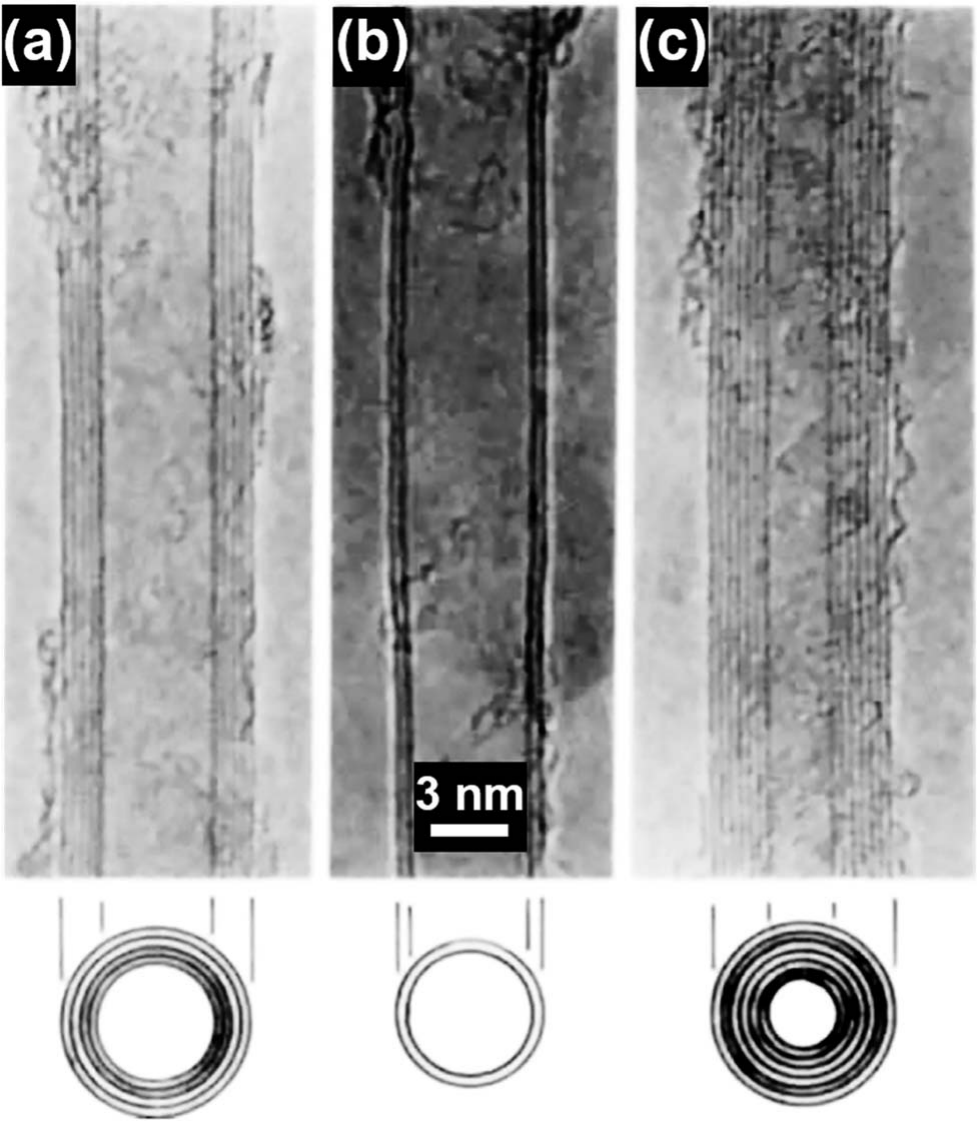
TEM images of (a) a five-wall CNT, diameter 6.7 nm, (b) a two-wall CNT, diameter 5.5 nm, and (c) a seven-wall CNT, diameter 6.5 nm synthesized by arc-discharge. Reproduced from ref. 1, with permission from Nature Publishing Group, 1991.

**Fig. 2:**
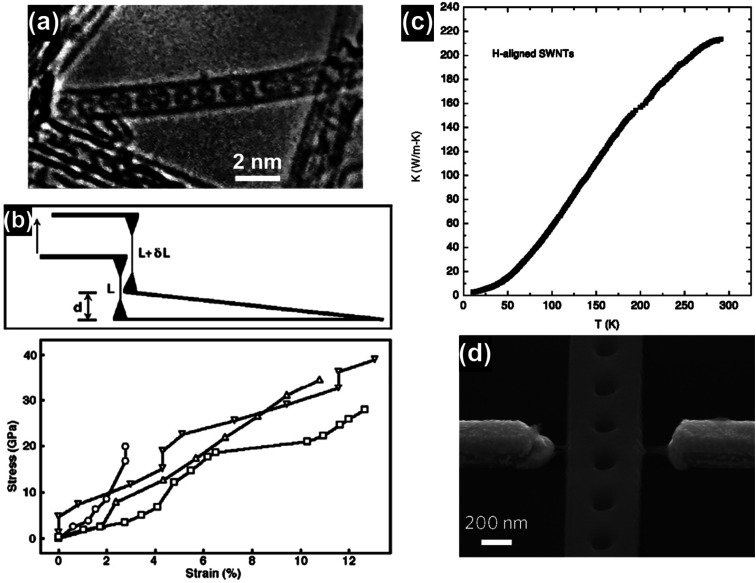
(a) A single-walled carbon nanotube filled with a row of C_60_ molecules along the tubule axis. Reproduced from ref. 2, with permission from Nature, 1998. (b) Stress–strain curves of individual MWNTs and the corresponding schematics of the test method. Reproduced from ref. 8, with permission from Elsevier, 2006. (c) Thermal conductivity of a bulk sample of SWNTs aligned by filtration under a strong magnetic field. Reproduced from ref. 10, with permission from Springer, 2002. (d) SEM image of a photonic crystal nanobeam cavity device coupled with a CNT light emitter. The waveguide at the center and two electrodes on its sides were bridged by a few SWNTs. Reproduced from ref. 11, with permission from Nature Publishing Group, 2016.

Carbon nanotubes (CNTs) have attracted significant research interest since their first discovery in 1991 and the representative CNT structures are shown in Fig. 1.^1^ Based on the number of the graphene walls, CNTs can be categorized into single-walled carbon nanotubes (SWNTs) and multi-walled carbon nanotubes (MWNTs). As a well-known allotrope of fullerene and graphene, CNTs represent a totally new structural dimension of nanocarbons. Extraordinary structural, mechanical, electrical, thermal and optical properties have been derived from the unique one-dimensional (1D) nanostructure of CNTs.^2–6^ For example, fullerenes can be encapsulated inside CNTs to form a new category of hybrid carbon nanomaterials, namely “peapods” (Fig. 2a), which may have distinct thermoelectric, optical and magnetic properties.^7^ A relatively high tensile modulus of 0.95 TPa and a tensile strength of 63 GPa can be achieved for arc-grown MWNTs, and values of ∼1 TPa and tens of GPa have been shown for high-quality SWNTs (Fig. 2b).^8^ Well-graphitized MWNTs with diameters from 8.5 nm to 18.5 nm may have resistivities ranging from 7.8 ± 1.0 ohm m to 117 ± 19 ohm m, depending on the local curvatures and defects in the tube walls.^9^ The thermal conductivity of SWNTs is measured to be higher than 200 W m^−1^ K^−1^, which is close to that of metals and an order of magnitude higher than that of crystalline graphite and diamonds (Fig. 2c).^10^ Moreover, high-speed nanoscale light emitters with narrow linewidths and high-speed modulation capabilities in the GHz range can be fabricated by combining SWNTs with nanophotonic cavities (Fig. 2d).^11^

**Fig. 3:**
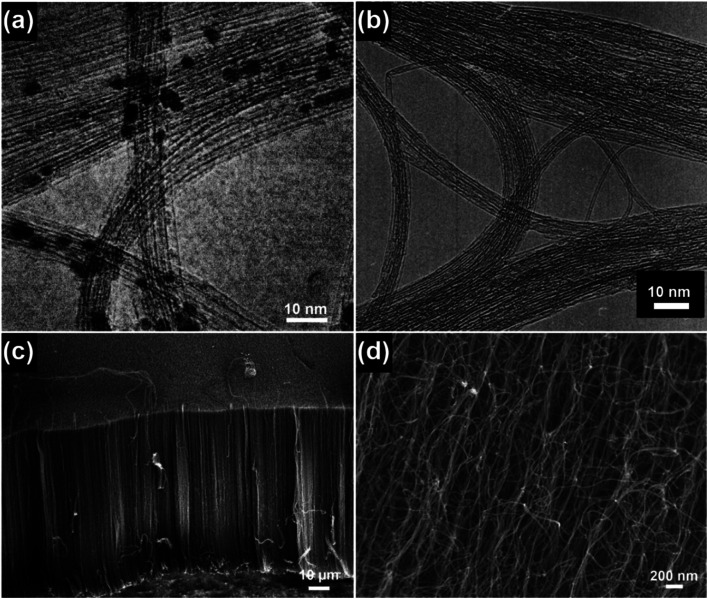
(a) TEM image of SWNTs produced by the HiPco method. Reproduced from ref. 12, with permission from Elsevier, 1999. (b) TEM image of SWNTs grown by catalytic chemical vapor deposition using ethanol as the carbon source. Reproduced from ref. 13, with permission from Elsevier, 2002. (c) SEM image of CNTs synthesized by using an atmospheric-pressure microwave torch. Reproduced from ref. 14, with permission from Elsevier, 2006. (d) SEM image of the bundle-type MWNTs synthesized by using a fluidized bed reactor. Reproduced from ref. 15, with permission from Elsevier, 2010.

The structural characteristics of CNTs are closely related to their synthesis conditions, such as the catalyst, temperature, pressure and feed gas. For example, when high temperatures (800–1200 °C) and high pressures (1–10 atm) are used, SWNTs can be synthesized by the so-called high-pressure carbon monoxide (HiPco) method, as shown in Fig. 3a.^12^ Upon switching the carbon source to alcohol, high-purity SWNTs with both no encapsulated metal nanoparticles and the presence of amorphous carbon impurities can be obtained at relatively low temperatures (700–800 °C) and low pressures (∼5 torr), as shown in Fig. 3b.^13^ By using an atmospheric-pressure microwave torch, dense and straight-standing MWNTs can be synthesized on a silicon (Si) substrate from 5 to 25 minutes (Fig. 3c).^14^ By using Co–Fe–Mo/Al_2_O_3_ as the catalyst, bundle-like MWNTs can be produced in high yield *via* a fluidized bed reactor (Fig. 3d).^15^ By using water-assisted chemical vapor deposition (CVD), a 1 mm tall SWNT forest can be directly grown on nickel (Ni) based alloys containing iron (Fe) and chromium (Cr) with a high selectivity of 95%.^16^ Despite their various synthesis conditions, SWNTs, few-walled carbon nanotubes (FWNTs) and MWNTs in total share several inherited structural features: their lengths can extend from a few to tens of micrometers, which go far beyond the nanoscale dimension and their diameters are set from a few to several tens of nanometers, which are within the nanoscale dimension. The nomenclature of the word “CNT” arises from the characteristic hollow-tube-like structure of these 1D nanocarbons, as graphene sheets are aligned parallel to the hollow-tube core and stacked to form the side-wall structure of the CNTs. The diameter of the hollow-tube core of CNTs is essentially dependent on the size of the catalyst nanoparticles and the growth conditions, *e.g.*, temperature and time. Provided that the growth is conducted below the liquefaction temperature of the metal catalyst, the tube diameter is directly proportional to the catalyst particle size.^17–19^ As the growth temperature is higher than the liquefaction temperature of the catalyst, the tube diameter may instead exhibit a universal Gauss-like distribution.^17,20^ The mechanical strength of the as-synthesized CNTs, *e.g.*, Young's modulus, is qualitatively related to the defects in the tube walls,^21–23^ and the tube diameter, number of walls and chirality may have little effects on Young's modulus.^24^ Similar to the case that one single CNT can be grown on one metallic nanoparticle, CNT bundles and forests can be grown on patterned arrays of metal nanoparticles as well. As the gaseous carbon species diffuse onto the surface of the metallic nanoparticles under the high growth temperature, CNTs may grow either by a “base-growth” or “tip-growth” pathway, depending on the synthesis techniques used. It was reported that large catalyst particles (>5 nm) favor the “tip-growth” mechanism to generate MWNTs while small catalyst particles (<5 nm) favor the “base-growth” mechanism to generate SWNTs and FWNTs (<7 walls).^25^

**Fig. 4:**
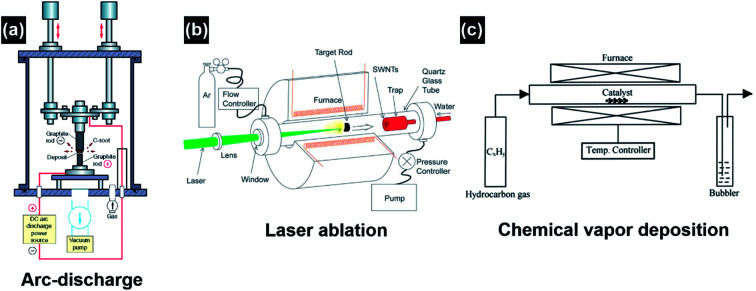
Schematic illustration of the experimental apparatuses of (a) arc-discharge, (b) laser ablation, and (c) thermal chemical vapor deposition for CNT synthesis. Reproduced from ref. 26, with permission from Elsevier, 2004.

The major conventional techniques involved in the synthesis of CNTs include arc-discharge, laser ablation and chemical vapor deposition (CVD) (Fig. 4).^26^ These techniques generally utilize a high-energy source (*e.g.*, high-voltage electric arc, laser beam, hot-filament, and plasma) to create highly dissociated or decomposed carbonaceous nanoclusters which subsequently diffuse onto the surface of catalyst nanoparticles and interact to induce CNT growth. In addition to the high-energy source, high growth temperatures (>873 K) are also required in these techniques to obtain CNTs with good crystallinity. For the arc-discharge and laser ablation techniques, the growth temperature can exceed 2000 K, leading to the formation of CNTs with high crystallinity, conductivity and mechanical strength.^27–29^ Inert-gas protection is also required during the arc-discharge and laser ablation processes, as free oxygen may react with the forming carbonaceous species and transform them into carbon dioxide at the high growth temperatures. Explosive gases such as acetylene, hydrogen and carbon monoxide are frequently used in the CVD growth of CNTs; in turn they add high explosion risks and raise serious safety concerns during the CNT growth process. The arc-discharge, laser ablation and CVD techniques use radiant and resistive heating mechanisms to grow CNTs, which means that the objects (*e.g.*, precursors, gases, substrates, and furnace walls) in the exposure range will be non-selectively heated simultaneously, and the optimization of the growth process and energy consumption is hard to achieve simultaneously. The CVD techniques also utilize an inductive heating mechanism, but the selectivity is limited to the conductive materials.^30^ Another problem associated with the non-selective heating process is the obscurity of the localized growth of the CNTs in the selected area. This problem is rather critical in the *in situ* site-selective fabrication of the CNT-based nanodevices on low-melting-point substrates, as the *in situ* growth of CNTs at the selected locations is speculated as the most efficient approach to establish nanoscale electrical contact and to manipulate the functions of nanoelectronics.^31–33^ The major drawbacks of conventional techniques are listed as follows:

(a) High-energy source requirement,

(b) Inert-gas protection,

(c) High growth temperature,

(d) High energy consumption and non-selective heating,

(e) Usage of explosive and toxic gases.

Different from conventional techniques, the heating process induced by microwave radiation does not rely on the external high voltage (*e.g.*, arc-discharge), high-energy laser beam (*e.g.*, laser ablation) and high-temperature furnace (*e.g.*, CVD). In contrast, it utilizes the internal microwave absorption properties of the substrate materials and catalysts to realize a selective and localized heating process for CNT growth.^34–37^ The selective heating for the CNT growth process can be realized by differentiating the microwave absorption properties, or more specifically the difference of the penetration depths between the substrate and the catalyst. For the absorbed microwave power in the unit volume of a material, there is:

1 *P* = 2π*fε*′′*E*^2^ = *σE*^2^

where *P* is the absorbed power per unit volume, *f* is the frequency, *ε*′′ is the complex permittivity, *σ* is the conductance and *E* is the electric field intensity. However, the value of *P* decreases as the penetration depth *d*_p_ decreases, and *d*_p_ is inversely proportional to the conductance of the material. For *d*_p_, there is:2
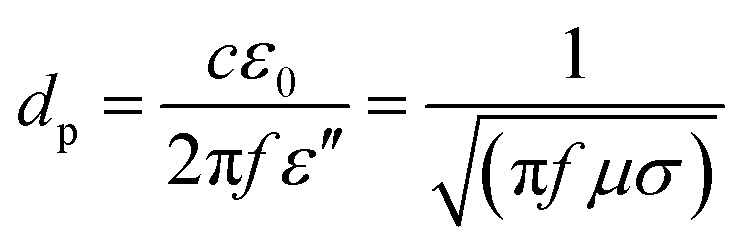
where *ε*_0_ is the dielectric constant and *μ* is the permeability of the material.

Therefore, the localized heating for CNT growth can be realized by choosing substrate materials with a low conductance and catalyst materials with a high conductance. For example, one can consider the case of Fe nanoparticle catalysts deposited on a SiO_2_ substrate. As microwave radiation is applied, the electromagnetic waves are preferentially absorbed by the catalyst nanoparticles and penetrate through the insulating substrate due to its large *d*_p_, resulting in the selective heating of the catalyst particles and the temperature rising in the local environment near the catalysts. On the other hand, the microwave transparent substrate can also dissipate the heat generated from the catalyst particles and maintain relatively low temperatures in the reaction vessel, making the low-temperature growth of CNT possible. Due to the volumetric, localized heating of the catalyst particles, the growth process of CNTs under microwave irradiation is very fast, which is typically in the range from 5 seconds (s) to 3 minutes (min).^34–36,38–41^

Besides the heating mechanism, microwave methods are substantially different from CVD methods in terms of the product, reactor design, and processing parameters. CVD methods, which are regarded as the most widely used techniques for CNT synthesis, are ideal to produce SWNTs, FWNTs and MWNTs with long lengths.^26,42–45^ With increasing growth temperatures from 500 °C to 1200 °C, the lengths of the CNTs synthesized by CVD methods can change from 0.1 μm to 10^5^ μm.^26^ On the contrary, microwave methods are more suitable to grow shorter CNTs with lengths ranging from 1 μm to 20 μm.^37,39,46,47^ To speak further, CVD methods are capable of producing SWNTs in the diameter range from 0.6–4.0 nm and MWNTs in the diameter range from 10–240 nm;^48^ while currently, microwave methods are able to generate MWNTs with diameters ranging from 10–200 nm.^37,39,41,46,47^ In this regard, different perspectives in applications can be envisioned for the CVD-grown CNTs and microwave-grown CNTs. The high aspect ratios of the CVD-grown CNTs make them ideal for the fabrication of polymer composites, conductive fillers, CNT fibers, *etc.*^49–51^ On the other hand, the lower aspect ratios of the microwave-grown CNTs make them suitable for surface modification and functionalization.^37,46,47^ The growth temperatures of CNTs in CVD techniques are typically in the range from 700 °C to 900 °C.^26^ Microwave methods may utilize higher growth temperatures of around 1000 °C, but the heating effect is highly localized in the proximity of the surface of the catalyst, resulting in a minimum increase of temperature in the surrounding environment.^46,47^ The CNT growth process of microwave methods can proceed by using mixtures of metallocenes and substrate materials, while the essential catalyst preparation steps in the CVD methods can be omitted. It thus makes microwave methods especially suitable for growing CNT brushes on thin, conductive and porous micro- and nano-dimensional substrates such as powders, fibers, graphene and MXenes.^46,47,52,53^

## 2. Fast synthesis of CNTs by microwave irradiation

### 2.1 Processes and instrumentation

**Fig. 5:**
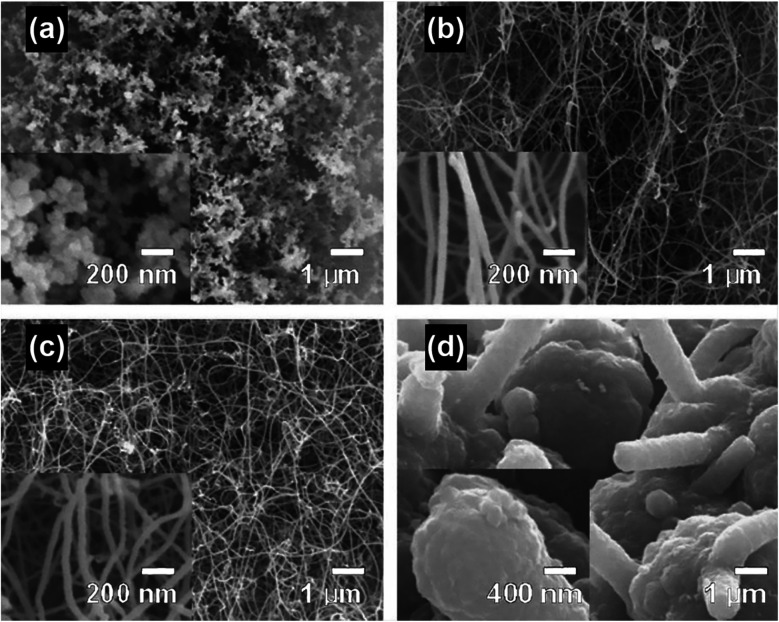
CNTs synthesized on activated carbon fiber cloth using microwave radiation for (a) 30 s, (b) 60 s, (c) 90 s and (d) 120 s. Reproduced from ref. 38, with permission from Elsevier, 2006.

By using microwave radiation, high-quality CNTs can be synthesized in a very fast manner. The reported synthesis time (*t*) for microwave radiation ranges from 5 s to 3 min, and *t* < 60 s is enough to produce long and entangled CNT forests according to most of the reported methods.^38–41,54^ Typical CNT forests prepared from microwave irradiation methods are shown in Fig. 5. In additional to the very short synthesis time, the post-synthesis processes of the microwave-generated CNTs such as separation and purification are also very simple and timesaving. Unlike the slow cooling steps involved in CVD as a result of the long time required for the heat dissipation of the furnace at elevated synthesis temperatures, cooling of the CNTs synthesized by microwave radiation can be instantaneously triggered as the microwave power ceases, as a result of the internal heating mechanism of microwave; moreover, the microwave transparent reaction vessels also help dissipate the heat generated by the microwave absorbing materials quickly, which in sum makes the cooling process feasible under ambient conditions and can be completed in a few minutes.^53–55^ After cooling, the as-obtained CNTs can then be separated from the carbon impurities by sonication and centrifugation.

**Fig. 6:**
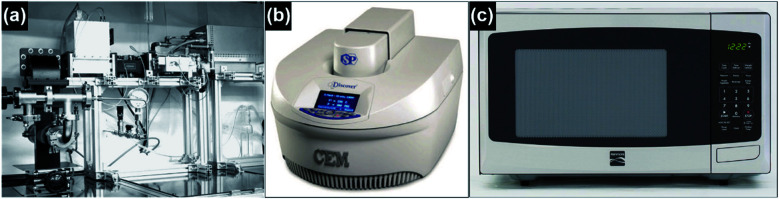
Schematic illustration of (a) the single-mode microwave system, reproduced from ref. 38, with permission from Elsevier, 2006; (b) laboratory microwave oven, reproduced from ref. 56, under the license of CC BY 4.0; and (c) domestic microwave oven, reproduced from ref. 57, under the license of CC BY 4.0.

Various instruments can be used to perform the synthesis of CNTs under microwave irradiation, including a single-mode microwave system, laboratory microwave oven and the domestic microwave oven, as shown in Fig. 6. High microwave power (2.45 GHz, up to 2000 watt), inert gas protection and pressure control can be equipped on the single-mode microwave system. In the meantime, single-mode reactors, monitoring cameras, and temperature and pressure controls are equipped in most of the laboratory microwave ovens.^56^ However, for the laboratory microwave ovens (*e.g.*, CEM discover®), the shape and volume of the reaction chamber are restricted, and this substantially limits the customization of reaction and large-scale production. On the other hand, the microwave ovens designed for large-scale synthesis (*e.g.*, Ultra Clave) are expensive and unsuitable for customized reactions. Such drawbacks can be circumvented by using domestic microwave ovens which provide reaction chambers with both high volumes that are compatible with high throughput synthesis and free space for customizing the experimental setups. Reaction vessels with different shapes can be placed into the domestic microwave oven and a variety of experimental configurations can be realized by the integration of vials, chambers and reactors, providing substantial capabilities of customization and control over the CNT growth process. Furthermore, the power and time control for the domestic microwave oven is facile and visual, making the parallel experiments highly reproducible. To further extend the synthesis functions of the domestic microwave oven, *e.g.*, inert gas protection and pressure and temperature control, special add-ons such as the Art Box™ furnace can be integrated inside the chamber to provide more sophisticated engineering control over the synthesis parameters to satisfy the needs for the fabrication of CNTs and the related nanocomposites.^58^ By customizing the microwave reactor with advanced functions for controlling the detailed parameters, a microwave synthesis system that is capable of producing CNTs with controlled side-wall structures and alignments, *e.g.*, SWNTs, FWNTs, and vertically aligned CNTs, can be realized. But in most cases, a pristine domestic microwave oven is sufficient for the synthesis of high-quality MWNTs for a wide range of applications.

### 2.2 Type and quality of the as-synthesized CNTs

**Fig. 7:**
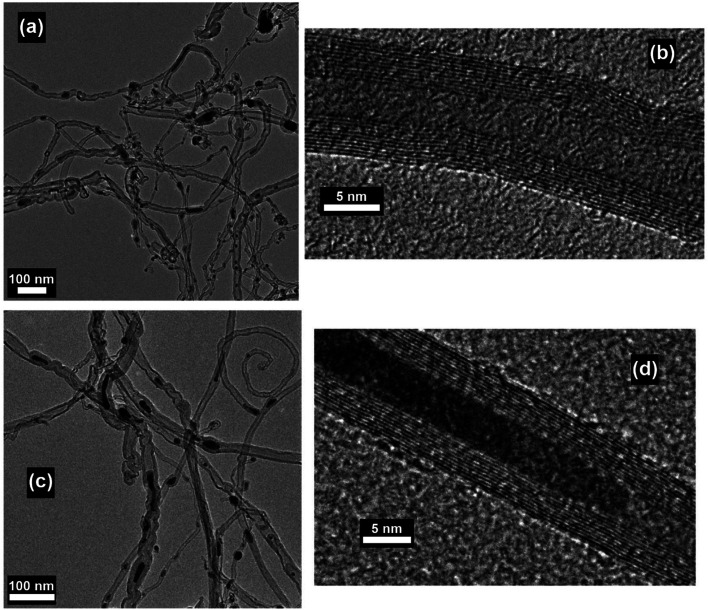
(a and c) TEM and (b and d) HRTEM images of MWNTs prepared by using ferrocene, graphite powder and carbon fiber as the precursor mixture. The synthesis was conducted in a commercial microwave oven (DEC18E2, ACP) and the irradiation time was 5 s. Reproduced from ref. 47, with permission from Elsevier, 2015.

**Fig. 8:**
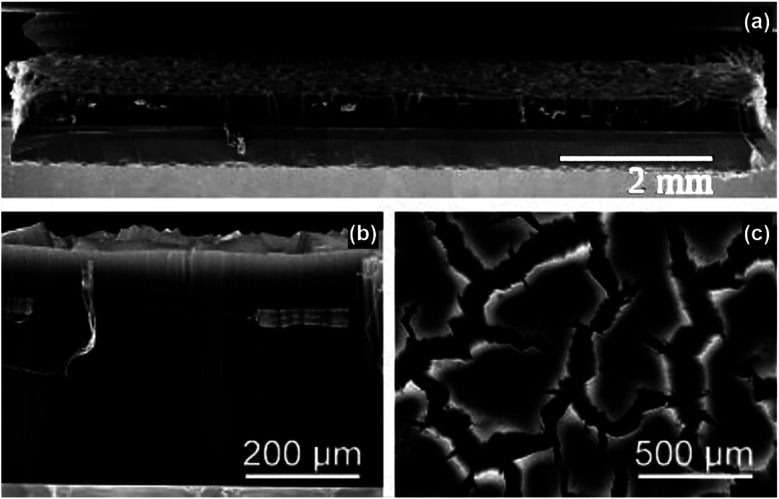
FE-SEM images of a 420 μm thick SWNT film on a 1 × 1 cm silicon wafer substrate prepared by microwave radiation: (a) the overview, (b) cross-sectional view and (c) top view. Reproduced from ref. 59, with permission from Wiley, 2005.

MWNTs can be readily grown at atmospheric pressure by using a domestic microwave oven and ferrocene as the precursor, and a conversion efficiency (from ferrocene to CNT) as high as 82 wt% can be achieved.^47^ The as-obtained MWNTs are observed as long, curly and entangled CNT bundles, as shown in Fig. 7a. The wall structure of the MWNTs is highly graphitized, and the lattice structure of the carbonized graphene layers can also be clearly observed (Fig. 7b). The growth direction of the graphene layers is aligned according to the residual catalyst nanoparticles that are stuck inside the cores of the MWNTs (Fig. 7c), and local curvatures are created by the nanoparticles along the tube axis which direct the growth of the MWNTs (Fig. 7d). By carefully tuning the growth conditions during microwave irradiation, *e.g.*, substrate temperature, reaction pressure, time, feed gas, microwave power and catalyst size, vertically aligned FWNT forests can be obtained (Fig. 8).^59^ However, the above-mentioned requirements involved in the synthesis control to generate the aligned FWNT forests will significantly complicate the instrument configuration, resulting in a microwave synthesis system that is similar to the CVD system. The MWNTs produced by the CVD methods typically show *I*_D_/*I*_G_ ratios in the range of 0.64–1.07.^60–63^ On the other hand, the MWNTs produced by the microwave methods show a relatively wide-range distribution of the *I*_D_/*I*_G_ ratios from 0.15 to 1.1, according to Raman spectroscopic characterization.^35,37–41,47,58,64^ The values of the *I*_D_/*I*_G_ ratio of the microwave-grown MWNTs are similar to, or smaller than, those of the CVD-grown MWNTs, indicating that the quality of the microwave-grown MWNTs is comparable to that of the CVD ones. The presence of the gaseous carbon sources and inert gases is found to enhance the degree of graphitization of the microwave-grown MWNTs, while at the same time decrease their defect densities, resulting in reduced *I*_D_/*I*_G_ ratios. For example, Odedairo *et al.* reported the growth of MWNT hybrids in a microwave reactor (SAIREM) with the addition of syngas as the carbon source and argon as the protective gas; *I*_D_/*I*_G_ ratios ranging from 0.15 to 0.37 can be achieved.^37^ Yoon *et al.* was able to produce MWNTs with an *I*_D_/*I*_G_ ratio of 0.41 by using activated carbon fiber as the carbon source and argon as the protective gas.^38^ Guo *et al.* also demonstrated that the presence of nitrogen protection can improve the *I*_D_/*I*_G_ ratio of the microwave-grown MWNTs from 1.04 to 0.854.^64^

### 2.3 The carbon yield

The yields of the MWNTs grown by microwave methods differ significantly and are strongly dependent on the growth conditions. By using acetylene as the carbon source, Hong *et al.* were able to obtain MWNTs on carbon black-supported metal catalysts, *i.e.*, Ni, Fe, and Co, in a commercial microwave oven with an applied power of 800 watt (W) and a reaction time of 30 min. Carbon yields between 200–350 wt% calculated based on the weight change of the catalyst before and after growth can be obtained, depending on the type of metal catalysts.^35^ By using syngas as the carbon source, Odedairo *et al.* managed to synthesize MWNT hybrids on ceria-supported bi-metal catalysts in a microwave reactor with a power of 500 W. The carbon yields of the process after 14 hours of growth differed from 0.61 g to 0.67 g based on the type of catalyst.^37^ By using ferrocene as the carbon source and catalyst, Nie *et al.* were able to synthesize MWNTs in 15–20 s in a domestic microwave oven with 1000 W applied power and a single carbon fiber as the heating filament. The carbon yield was calculated to be 40 wt% based on the weight of ferrocene.^41^ Since the theoretical carbon content in ferrocene is approximately 64.5 wt%, the conversion efficiency of the carbon element in ferrocene to MWNTs is approximately 62% in this case. By using ferrocene as the carbon source, graphite as the microwave absorber and carbon fiber as the heating filament, Bajpai *et al.* were able to grow MWNTs in a commercial microwave oven (DEC18E2, ACP) with an applied power of 1800 W and a time of 5 s. The carbon yield based on the weight of the precursor mixture was measured to be 26 ± 5 wt%. In this case, the conversion efficiency of the carbon element in ferrocene to MWNTs is approximately 66.5–98.2%.^47^ However, the higher carbon yields in the latter case can be attributed to the addition of graphite which effectively absorbed the microwave power and generated substantial heat to trigger the fast decomposition of ferrocene for CNT growth.

The fast and cost-effective CNT synthesis process enabled by the domestic microwave oven can easily generate MWNTs with appropriate functions (*e.g.*, crystallinity, conductivity, and aspect ratio) in high yields, with low instrumental and precursor requirements. The as-obtained MWNTs can be further used in a wide-range of applications including fillers, field emission displays, transistors and lithium-ion batteries.^64–67^

## 3. Fast synthesis of CNTs on different substrates by microwave irradiation

### 3.1 Processes, substrates and products

**Fig. 9:**
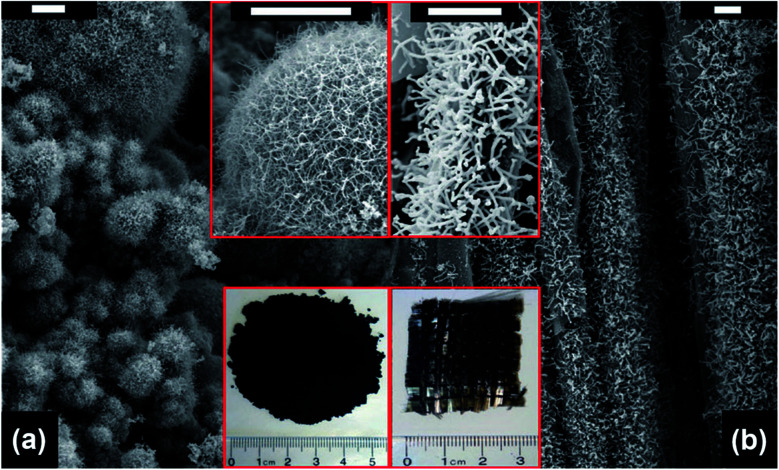
SEM images of CNT brushes grown on (a) fly ash and (b) glass fiber fabrics. The insets show the magnified images of the CNT brushes (upper site) and the photographs of the as-obtained products (bottom site), respectively. Scale bar: 5 μm. Reproduced from ref. 46, with permission from the Royal Society of Chemistry, 2011.

**Fig. 10:**
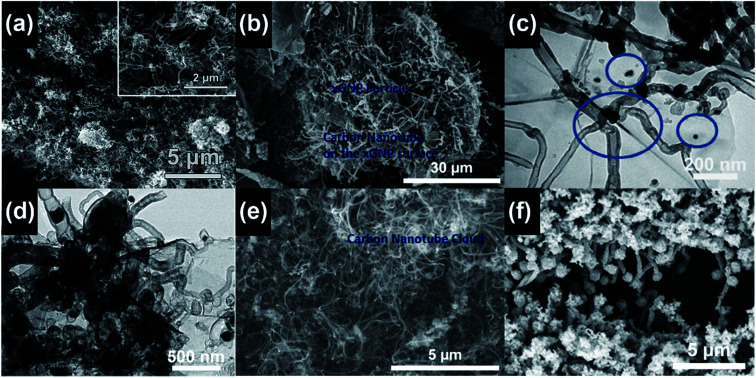
(a) CNT brushes grown on a Ti_3_C_2_ MXene after three sequential cycles of 900 W microwave power and 40 s processing time. Reproduced from ref. 52, with permission from the Royal Society of Chemistry, 2018. (b) CNT brushes grown on exfoliated graphite nanoplatelets (xGNPs) with 1100 W microwave power and 10 s processing time. Reproduced from ref. 64, with permission from Elsevier, 2017. (c) CNT bundles grown on the graphene surface with 700 W microwave power and 2 min processing time. The blue circles mark the locations of the Fe nanoparticle catalysts. Reproduced from ref. 68, with permission from Elsevier, 2015. (d) CNT brushes grown on graphene oxide and zeolitic imidazolate framework substrates with 700 W microwave power and 30 s processing time. Reproduced from ref. 71, with permission from Elsevier, 2019. (e) CNT brushes grown on graphite powders with 1100 W microwave power and 15 s processing time. Reproduced from ref. 64, with permission from Elsevier, 2017. (f) CNT brushes grown on the Ppy coated woven Kevlar fibers by microwave radiation. Reproduced from ref. 70, under the license of CC BY 4.0.

By using the microwave radiation, CNT brushes can be directly grown on different substrates *in situ*, such as graphite, graphene, MXenes, Kevlar, carbon fiber and fly ash.^46,47,64,68–70^ In this regard, the domestic microwave oven can be used to realize the CNT growth on the various substrates and materials at atmospheric pressure without an external furnace. For example, MWNT brushes were grown on fly ash and glass fiber fabrics by using a domestic microwave oven (Fig. 9). The experimental set-ups were very simple and only required the mixing of the polypyrrole (Ppy) coated fly ash (or glass fiber fabrics) with ferrocene, and then 1250 W microwave power was applied on the mixture for 30 s by using a domestic microwave oven. The CNT growth process was completed in a single glass vial and no external heating devices and pressure vessels were required. However, intensive absorption of the microwave radiation by the mixture can increase its temperature to reach the growth temperature in a few seconds; the pressure inside the reaction vial is also increased quickly as ferrocene is decomposed into abundant gaseous carbonaceous compounds by the dielectric heating of microwave. Besides the fast and facile growth, CNTs can be grown on substrates of different dimensional characteristics, from nanosheets and micro-fibers to macro-scale surfaces. For example, CNT brushes can be synthesized on the surface of 2D nanomaterials, such as MXenes, exfoliated graphite nanoplatelets and graphene (Fig. 10a–c); they can also grow on micrometer-size materials, such as ceramic powders, graphite powders, Kevlar fibers and glass fibers (Fig. 10d–f) and on macro-scale materials' surface such as glass, Teflon, and polycarbonate.^34–36^ Besides the growth of CNT brushes on different substrates, simultaneous functionalization of the CNTs during growth can also be achieved by the introduction of functionalization reagents into the precursor mixture. For example, nitrogen-doped CNT brushes can be obtained by adding azobis(cyclohexanecarbonitrile).^68^ Metal-decorated (*e.g.*, Fe and Pd) magnetic CNT brushes were obtained by the incorporation of iron compounds into the precursor mixture.^68,70,71^ Metal oxides/CNT brushes/Fe hybrid nanostructures were also fabricated by the oxidation of MXenes under microwave irradiation.^52,69,72^

### 3.2 The growth mechanisms

Based on the intrinsic microwave absorption properties of the substrate, the CNT brushes can undergo either a “direct growth” pathway or a “medium growth” pathway. For the microwave absorbing substrates, such as Ppy, graphite, graphene oxide and carbon fiber, the direct growth pathway is favorable since the substrate itself can effectively absorb microwaves and generate sufficient heat to reach high local temperatures on the surface to trigger growth. However, when microwave transparent substrates are used, such as Kevlar, Teflon, fly ash and glass fiber, the medium growth pathway is more favorable since the substrate itself does not absorb microwaves and cannot generate enough heat under microwave irradiation. In this regard, an additional layer of microwave absorbing materials (*e.g.*, polypyrrole, graphene, and metal sulfides) can be introduced onto the surface of the substrates *via* a coating process, such as dip coating, spray coating and sputter coating. The microwave absorbing materials, *i.e.*, the microwave absorbers shown in Table 1, are mediated between the microwave-transparent substrates and catalyst nanoparticles. On one hand, they can serve as the binder to combine both the substrate and the catalyst; on the other hand, they vigorously absorb the microwave power and generate substantial heat, raising the temperature of the precursor blend (*e.g.*, the mixture of the catalyst, microwave absorber and substrate material) to the appropriate range that is suitable for the growth of CNTs. In several cases, the microwave absorbers also served as the carbon source to support CNT growth.^38,58^ This phenomenon can be attributed to the intimate deposition of the catalyst nanoparticles on the surface of the microwave absorber, and the recrystallization of the amorphous carbon inside the microwave absorber under the growth conditions. In both of the “direct growth” and “medium growth” pathways, metal compounds such as ferrocene, iron acetate and cobalt sulfide are often selected as the catalysts due to their high dispersibility and reactivity.^34–36,46,52,64–72^ Before microwave irradiation, the catalyst is either directly mixed with the substrate materials or coated on the substrate surface (in the direct growth pathway), or can be mixed with/coated on the microwave absorbing material-coated substrate (in the medium growth pathway). During microwave irradiation, the metal compounds quickly decompose into metal nanoparticles, serving as the new catalytic centers for CNT growth; low-molecular-weight hydrocarbons are also generated by the decomposition of the metal compounds, and they can serve as the primary carbon source to support the growth. Closed vessels are more favorable than open ones as the container for the microwave growth of CNTs. Closed vessels, *e.g.*, capped scintillation vials, can effectively confine the gaseous carbonaceous species generated by the decomposition of the metal compounds, resulting in a higher carbon concentration and internal pressure within the vessels to support CNT growth. Domestic and commercial microwave ovens with powers ranging from 800 W to 2000 W are suitable for the fast growth CNT brushes on various substrates. A detailed comparison regarding the CNT growth parameters on different substrates is provided in Table 1.

**Table 1:** Fast synthesis of CNTs *via* domestic microwave ovens: detailed information on the catalysts, carbon sources, substrates, processing parameters and growth conditions is shown

Reference	Power	Time	Catalyst	Carbon source	Substrate	Microwave absorber	Condition	Product
38	400 W	30–120 s	Fe	Activated carbon fiber	Activated carbon fiber	Activated carbon fiber	Argon	MWNT
39	800 W	20–40 s	Ferrocene	Ferrocene	Ferrocene	Cu wires	Atmospheric	MWNT
40	800 W	30 s	Ferrocene	Ferrocene	Graphite	Graphite	Atmospheric	MWNT
41	1000 W	15–20 s	Ferrocene	Ferrocene	Ferrocene	Carbon fiber	Atmospheric	MWNT
46	1250 W	15–30 s	Ferrocene	Ferrocene	Fly ash	Ppy coating	Atmospheric	MWNT coated fly ash
47	1800 W	5 s	Ferrocene	Ferrocene	Graphite	Graphite, carbon fiber	Atmospheric	MWNT
52	900 W	40 s	Ferrocene	Ferrocene	Ti_3_C_2_	Ti_3_C_2_, carbon fiber	Atmospheric	CNT@Ti_3_C_2_ hybrids
53	700 W	180 s	Ferrocene	Ferrocene, azodicarbonamide	Reduced graphene oxide (rGO)	rGO	Atmospheric	3D rGO/CNT/Fe
54	1250 W	10–20 s	Ferrocene	Ferrocene	Poly(lactic-*co*-glycolic acid) (PLGA)	Ppy	Atmospheric	CNT-Fe-PLGA particles
64	1100 W	10 s	Ferrocene	Ferrocene	Exfoliated graphite nanoplatelet (xGNP)	xGNP, carbon fiber	Atmospheric	MWNT coated graphite
65	810 W	3 min	Palladium acetate	Palladium acetate	Graphite oxide	Graphite oxide	Atmospheric	Pd-CNT-rGO composite
66	1000 W	3–12 min	Cobalt acetate	Cobalt acetate	Graphite	Graphite	Vacuum (10^−4^ torr)	MWNT sponges
67	N/A	190 s	Ferrocene	Ferrocene, azodicarbonamide	Acetylacetonate	Acetylacetonate	Atmospheric	3D CNT/N-doped rGO/Fe hybrids
68	700 W	2 min	Fe	Azobis(cyclohexanecarbonitrile)	Graphene	Graphene	Atmospheric	Graphene-CNT hybrids
69	900 W	20 s	Ferrocene	Ferrocene	Ti_3_C_2_ (MXene)	Ti_3_C_2_	Atmospheric	3D metal oxide/carbon nanotube/iron hybrids
70	1000 W	N/A	Ferrocene	Ferrocene	Kevlar fiber	Ppy coating	Atmospheric	Fe-CNT/Ppy-coated Kevlar fiber
71	700 W	30 s	Co	Zeolitic imidazolate frameworks	Graphene oxide	Graphene oxide	Atmospheric	Graphene/Co/N-doped CNT hybrids

## 4. Selective heating and growth of CNTs under microwave irradiation

### 4.1 Growth of CNTs at low temperatures and atmospheric pressure

**Fig. 11:**
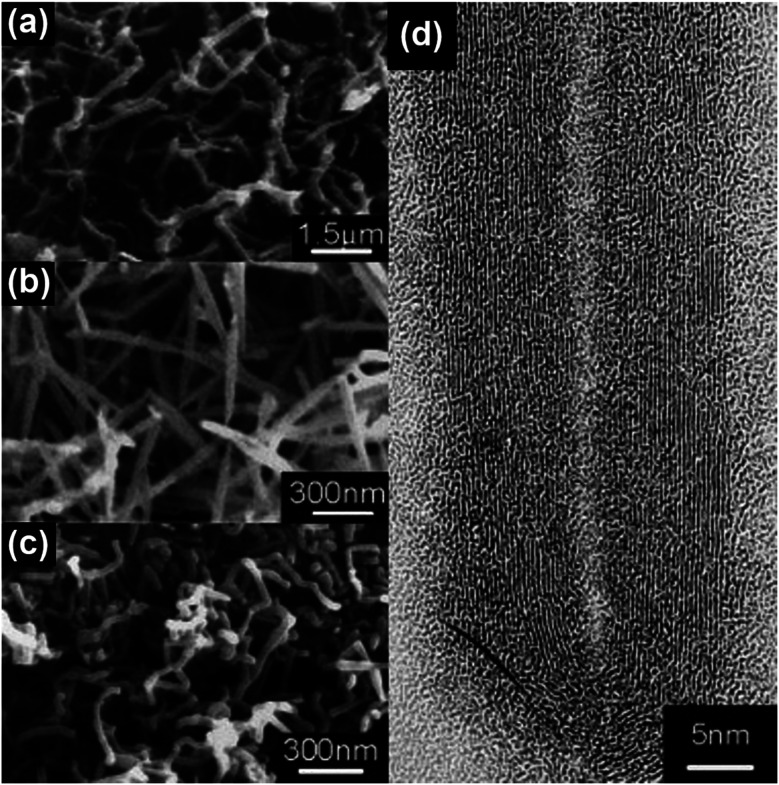
SEM images of CNT brushes grown on (a) polycarbonate and (b and c) Teflon substrates with (a) cobalt naphthenate and (b and c) Co_9_S_8_ as the catalysts. (d) HRTEM image of the MWNT grown in (b). Reproduced from ref. 35, with permission from Wiley, 2003.

**Fig. 12:**
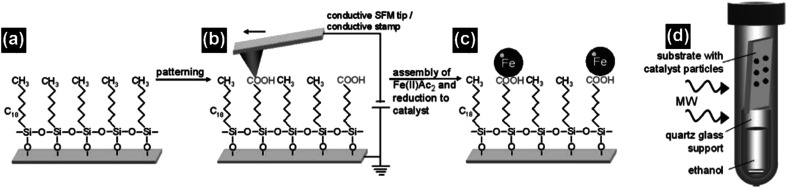
(a and b) The *n*-octadecyltrichlorosilane monolayer was patterned using a conductive scanning force microscopy tip, which converted its methyl groups into carboxylic groups. (c) The carboxylic groups can act as the anchor sites for the ferrous cations, which were subsequently reduced to form iron nanoparticles as the catalysts. (d) The iron catalyst loaded *n*-OTS layer on a silicon substrate was placed on top of a quartz glass support filled with ethanol in a pressure vessel and irradiated with microwaves to grow CNTs. Reproduced from ref. 36, with permission from Wiley, 2009.

One of the major advantages of the synthesis of CNTs by microwave irradiation is the low synthesis temperature, which can enable the *in situ* growth of CNTs on low-melting-point substrates, *e.g.*, polytetrafluoroethylene (PTFE), polycarbonate and organosilane monolayers, through a selective heating pathway that preferentially heats the catalyst particles.^34–36^ To grow CNTs on low-melting-point substrates, appropriate selection of the catalyst, microwave power, time and carbon source is required. For example, no CNTs can be formed on the PTFE substrate under microwave irradiation if cobalt is used solely as the catalyst.^34^ This phenomenon can be attributed to the low microwave absorption properties of the PTFE substrate which dissipate the heat generated by Co catalyst nanoparticles under microwave irradiation, and the limited microwave absorption properties and catalytic activity of Co. In this case, a new kind of catalyst, metal sulfide nanoparticles, can be used to grow CNTs on low-melting point substrates such as PTFE. Compared with pure metal nanoparticles, metal sulfide nanoparticles may have higher microwave absorption properties, lower melting temperatures, and higher surface catalytic activities.^73–75^ These characteristics of metal sulfides are more favorable for the growth of CNTs. By using Co_9_S_8_ as the catalyst, CNTs with well-defined graphitized wall structures were successfully synthesized on low-melting-point substrates such as polycarbonate and Teflon by microwave irradiation (800 W, 2.45 GHz) within 3 minutes (Fig. 11). Patterned CNT growth can also be realized on conductive substrates such as silicon by the selective heating mechanism. In this case, the catalyst nanoparticles should be carefully deposited and patterned on the substrate before the initiation of the catalytic growth process. Patterning of the catalyst nanoparticles can be achieved by either monolayer self-assembly, phase separation of block co-polymers or photolithography.^36,76,77^ For example, iron nanoparticles were patterned on the silicon surface by using a self-assembled monolayer of *n*-octadecyltrichlorosilane (OTS). The surface of the OTS layer was precisely oxidized using a conductive scanning force microscopy tip and the formed carboxylic groups that could bond with ferrous iron cations (Fe^2+^). Afterwards a hydrazine vapor reduction process was applied on the OTS surface and patterned Fe nanoparticles were obtained. A microwave power of 300 W was used to grow CNTs on the Fe loaded and OTS coated silicon substrate. At this time, the Fe nanoparticles were heated by microwave irradiation selectively to induce CNT growth. As a result of the selective-heating mechanism, the growth process was able to proceed at very low environmental temperatures (130–150 °C) and a short time (3 minutes) (Fig. 12).

### 4.2 The mechanism of selective heating and the role of metal catalysts

The selective heating of microwaves can realize the growth of CNTs at low temperatures and atmospheric pressure. It will therefore make the *in situ* growth of CNTs on plastics and sensitive electronics such as circuits possible. These CNT-containing composites can be further used in flexible electronics, sensors and field-effect transistors. The selective heating mechanism of microwaves is based on the sharp difference between the microwave absorption properties of the catalysts and the substrates, which would result in the preferential absorption of the microwave power by the catalysts and raise the temperatures near the catalyst's surface. Therefore, the effect of selective heating induced by the microwaves can be maximized when the catalyst is microwave-absorbing and the substrate is microwave-transparent. It can be minimized when both the catalyst and substrate are microwave-absorbing. However, to improve the growth conditions of CNTs, it is better to choose metallic nanoparticles with higher microwave absorption properties for achieving higher temperatures on the surface of the nanoparticles.

Due to the effect of heat dissipation induced by the microwave-transparent substrate, it is hard for the pure metal nanoparticles to reach the appropriate temperature range for CNT growth on their surface under the electromagnetic field of microwaves.^34–36^ To improve the microwave-absorption properties of the metal nanoparticles and make it sufficient to sustain CNT growth, several strategies can be employed besides sulfurization, including porosification, morphology engineering and particle size control.^78–83^ For example, T. Liu *et al.* reported that the microporous Co nanoparticles derived from Co–Al alloy nanoparticles can have enhanced microwave absorption properties in terms of low reflection loss (RL) and wide absorption bandwidth.^78^ By converting the Ni compounds into a 3D flower-like structure, D. Liu *et al.* were able to obtain Ni microspheres with enhanced microwave absorption properties.^80^ Wen *et al.* also proposed that the microwave absorption properties of cobalt powders can be enhanced by changing their morphologies from spherical to flake-like.^81^ By controlling the sizes of the ferrite particles, Panwar *et al.* demonstrated that minimum RL can be obtained by changing the particle size ranges from 200–250 nm to 20–30 nm.^83^

## 5. Conclusions and future work

The synthesis and growth of carbon nanotubes by using commercial or domestic microwave ovens present a new pathway for the fabrication of CNTs and related materials. Microwave methods feature very short processing time, wide-range adaptability to different substrates, non-solvent reaction and highly efficient selective-heating mechanism. They can significantly reduce the processing requirements for CNT growth, *e.g.*, time, equipment, feed gas and cost. The unique selective-heating mechanism of microwave can realize the growth of CNTs at low temperature and atmospheric pressure. By using these advantages, *in situ* growth of CNTs at selected locations on silicon wafers and chips, or low-melting-point substrates such as Teflon, can be achieved by microwave methods, and they can be further utilized to fabricate and manipulate CNT-based nanoelectronics, circuits and sensors *in situ*. On the other hand, microwave methods have been proven to be highly adaptive to produce various CNT-based nanocomposites. Different kinds of carbon sources, catalysts, substrates and dopants can be simultaneously combined and irradiated in a domestic microwave oven which is turned into a simple but effective plasma reactor to synthesize new CNT-based composites and functionalized CNTs suitable for a wide-range of applications from lithium-ion batteries, field-effect transistors, and thermoelectronics to photonics.

However, current CNTs produced by microwave methods suffer from drawbacks such as high defect density, random orientation and low structural homogeneity. To address these problems, the future microwave CNT synthesis techniques should strike a balance between the growth rate and microstructural control, *e.g.*, to use longer synthesis time and lower microwave powers for more homogeneous growth. To achieve low-temperature CNT growth at atmospheric pressure, sulfurized metal nanoparticles such as Co_9_S_8_ and FeS_2_ can be used as high-reactivity catalysts instead of conventional metallic compounds and nanoparticles. Porosity, morphology and particle size engineering can also be applied for pure metal nanoparticles to enhance their microwave absorption properties. The defect density of the as-grown CNTs can be effectively lowered by the addition of reducing reagents while their orientation can be improved by using low-molecular-weight hydrocarbons such as methane, ethane and ethanol as the precursors. After all, the efforts for improving the microwave methods would eventually afford fast, large-scale, low-cost synthesis of high-quality oriented CNT brushes that are suitable for a wide range of applications.

## Conflicts of interest

The authors declare no conflict of interest.

## Supplementary Material
